# Association between Air Pollution and Physical Activity and Sedentary Behavior among Adults Aged 60 Years or Older in China: A Cross-Sectional Study

**DOI:** 10.3390/ijerph20032352

**Published:** 2023-01-28

**Authors:** Bing Zhan, Qiurui Wang, Zhixiong Zhou, Xiaotian Li, Hongjun Yu, Bingzhao Li, Mingxia Liao

**Affiliations:** 1 School of Sport Management and Communication, Capital University of Physical Education and Sports, Beijing 100091, China; 2 Institute of Artificial Intelligence in Sports, Capital University of Physical Education and Sports, Beijing 100091, China; 3 School of Physical Education and Coaching Science, Capital University of Physical Education and Sports, Beijing 100091, China; 4 School of Recreation and Community Sport, Capital University of Physical Education and Sports, Beijing 100091, China; 5 Department of Physical Education, Tsinghua University, Beijing 100084, China; 6 Department of Mathematics and Statistics, Beijing Institute of Technology, Beijing 100081, China

**Keywords:** AQI, older adults, exercise, outdoor

## Abstract

Background: Exposure to air pollution is associated with an increased risk of all-cause mortality in older adults. Promoting physical activity (PA) and avoiding sedentary behavior (SB) serve as key strategies to maintain and improve human health. However, ambient air pollution can adversely affect PA and SB, increasing the risks of health problems. This study aimed to visualize national spatial patterns of average AQI concentration, PA, and SB distributions and to examine the associations between air pollution and PA and SB in a national sample of Chinese older adults aged 60 years or older. Methods: We analyzed the data of the China Longitudinal Aging Social Survey 2020 (CLASS 2020), which sampled 11,399 older men and women from 30 cities in China. Moderate, vigorous, and light PA and SB were measured using the Chinese version of the International Physical Activity Questionnaire-Short Form (IPAQ-C). The environmental measures included the average hourly air quality index (AQI), PM_2.5_, PM_10_, and NO_2_ (µg/m^3^). The data were analyzed using multivariable linear regression. Results: Increases in the standard deviations (±SD) of AQI, PM_2.5_, PM_10_, and NO_2_ concentrations were associated with decreases in MVPA per week of −2.34 (95%CI = −3.36, −1.32), −2.58 (95%CI = −3.61, −1.55), −1.96 (95%CI = −3.05, −0.08), and −1.19 (95%CI = −2.06, −0.31) and decreases in LPA per week of −6.06 (95%CI = −7.15, −4.97), −4.86 (95%CI = −5.88, −3.85), −4.78 (95%CI = −5.89, −3.68), and −4.59 (95%CI = −5.57, −3.61) h/week, respectively. Increases in one SD of AQI, PM_2.5_, PM_10_, and NO_2_ were associated with increases in SB per week of 1.32 (95%CI = 0.77, 1.88), 0.62 (95%CI = 0.09, 1.14), 1.03 (95%CI = 0.48, 1.59), and 0.98 (95%CI = 0.46, 1.49) h/week, respectively. Conclusions: The spatial distributions of the average AQI concentration, MVPA, LPA, and SB are useful and allow environmental and health policymakers to identify the areas with the highest priority air pollution environmental equality concerns. AQI was positively associated with MVPA and LPA, and it was negatively associated with SB among older adults. AQI, PM_2.5_, PM_10_, and NO_2_ were hardly associated with women’s average time spent engaged in MVPA. Region-specific and multi-level health policy options are needed to reduce ambient air pollution by taking different types of pollutants into account in order to avoid changes in PA and SB in this population, especially in locations with high air pollution concentrations.

## 1. Introduction

As life expectancy increases, population aging has become an important focus of global health. Exposure to air pollution has detrimental effects on human health and health-related behaviors, associated with increased risk factors for non-communicable diseases and all-cause mortality in older adults on a global scale [1,2]. Adequate evidence shows that older adults (≥65 years old) are more susceptible to air-pollution-induced health effects due to decreased physiological, metabolic, and compensatory processes and a greater incidence of cardiovascular and respiratory diseases [3]. In the U.S. Medicare population from 2000 to 2012, short-term exposure to particulate matter of less than 2.5 µm (PM_2.5_) and a warm-season ozone were found to be significantly associated with an increased risk of mortality among older adults [4]. Particulate air pollution has been found to be associated with abnormalities in cardiac autonomic control [5], respiratory function [6], and increased mortality in the Medicare aging population [7]. The risk factors for chronic diseases are mainly caused by an unhealthy and sedentary lifestyle [8]. Thus, a worldwide increase in interest in health-enhancing physical activity has been observed [9]. Physical activity has protective and preventive effects against chronic diseases and the risk of disease progression. However, the physical activity levels of adults may decrease during high air pollution periods or in highly polluted environments [10]. Previous research has also revealed that exercising in environments with high levels of air pollution can increase the risk of health problems, ranging from asthma attacks to heart or lung pathologies [11].

Both physical activity and sedentary behavior are independently linked to chronic diseases and conditions. Physical activity (PA) is an effective modality to reduce or prevent functional decline associated with aging [12]. Significantly, reductions in risk factors associated with disease states (e.g., heart disease, diabetes, and cancer) improve health status and increase life expectancy. Sedentary behavior (SB) includes any behavior with low energy expenditure (≤1.5 metabolic equivalents (METs)), such as TV viewing; computer use; and sitting during commuting, in the workplace, and during leisure time [13]. Many studies of the adverse effects of low PA have revealed a complex, multifaceted relationship between physical work, energy expenditure, and health [14]. Compared with other age groups, adults aged 50 to 64 years and 65 years or older are the most sedentary in the U.S. and Europe [15,16]. A global PA assessment in more than 60 countries found that older adults had the highest prevalence of reporting a daily sitting time of 4 or more hours [17]. Additional evidence has reported a relationship between SB and all-cause mortality, cardiovascular disease, and cancer among older adults [11,18]. Much research on the adverse effects of ambient or outdoor air pollution on health has been documented [19,20,21]. An et al. systematically reviewed ten studies linking ambient pollution to PA and SB, showing the inverse and positive associations between air pollution and PA and SB in adults [22]. A recent systematic review has linked air pollution to PA-related adverse associations [23]. In summary, from the global perspective, air pollution may influence outdoor human behaviors, and significant research exists regarding the health benefits and disease prevention of increasing PA and reducing SB for older adults. Since effective prevention is necessary to keep the older adult population in good health for as long as possible, it is essential to understand the physical activity behaviors associated with exposure to air pollution risk factors among this large population group.

In China, the total number of older people aged 60 years and over reached approximately 267 million, or 18.9% of the entire population, in 2021. The proportion of older people is projected to increase to 26% by 2050, exceeding that of most European countries. Luo et al. reported that, among 4134 older adults aged 60–80 years, the proportion of older adults meeting the sedentary time recommended in the 24-h Movement Guidelines is only 12.93% in China [24]. Increasing physical activity and reducing sedentary behavior are cost-effective ways to improve health. In China, the average 24 h PM_2.5_ concentration has been found to exceed the World Health Organization (WHO) air quality guidelines (AQGs) and the new AQGs by 75.4% and 51.1% person-days, respectively [25]. Exposure to ambient particulate matter increases the risks of cardiovascular diseases, myocardial infarction, stroke, lung cancer, respiratory diseases, and all-cause mortality in China [26,27]. Recent studies have estimated that the air pollution in 272 Chinese cities is significantly associated with cause-specific mortality [28,29]. Yu et al. found that air pollution discouraged physical activity and increased sedentary behavior among 2235 freshman students living in Beijing [30]. However, less is known regarding air pollution’s impact on health behaviors, notably PA (moderate-to-vigorous intensity PA and LPA) and SB among susceptible sub-populations, such as older adults [31]. Few studies have examined air pollution’s impact on PA and SB among older adults in China. Among the few previous studies conducted in China, Yu et al. focused on the relationship between PM_2.5_ and PA among university retirees living in Beijing [32]. Huang explored various mediators (PM_2.5_, PA, and BMI) to determine the association between greenness and hypertension from 2007 to 2010 [33]. Sun showed that mortality risks among older Chinese adults living in Hong Kong were inversely associated with a higher volume of PA and were positively associated with long-term exposure to PM_2.5_ [34]. In summary, PM_2.5_ and PA and SB have been found to be adversely and positively associated, respectively, among older people in local areas of China.

The AQI is an index for reporting daily air quality, including the air conditions and alerts for associated health concerns. The China Meteorological Administration calculates the AQI for five measures of air quality: ozone, particulate matter (<2.5 µg and <10 µg), carbon monoxide, sulfur dioxide, and nitrogen dioxide. However, the few number of studies examining the association between air pollution and PA and SB indicate three significant gaps in the current scientific literature that warrant further investigation. First, studies regarding the association between air pollution and PA or SB among older Chinese adults should be extended. Second, previous studies have exclusively focused on the impact of PM_2.5_ on PA and health among older adults. However, to the best of our knowledge, no research has examined the relationship between AQI and different air pollutants (PM_2.5_, PM_10_, and NO_2_) and PA and SB among older adults. Third, most existing studies have focused on urban Chinese older adults living in a specific province in China, namely, Beijing or Hong Kong. Studies have yet to cover a large-scale sample of the whole country.

This study uses cross-sectional data to examine the relationship between air pollution, PA (moderate-to-vigorous intensity PA and light PA), and SB among Chinese older adults from 476 villages within 30 provinces in 2020. Our specific objectives were as follows: (a) to create a visual representation of the national spatial patterns of average AQI concentration, PA, and SB distributions in seven administrative regions of China; (b) to determine the independent relationships between PM_2.5_, PM_10_, and nitrogen dioxide (NO_2_) and three variables (moderate-to-vigorous and light PA and SB) among Chinese older adults; and (c) to assess the differences between the above associations.

## 2. Materials and Methods

### 2.1. Participants

This article’s data were obtained from the 2020 China Longitudinal Aging Social Survey (CLASS 2020). The CLASS questionnaire began in 2014, and it was followed up every two years. The physical activity component of the survey was introduced in 2016. The survey was conducted by the Chinese Investigation and Statistics Center (NSRC) of Renming University from 1 March to 30 November 2020. CLASS 2020 is a household survey aiming to understand older Chinese adults’ socio-economic backgrounds in order to identify the various challenges experienced during the aging process. The survey adopted a multi-stage, random clustering process to sample more than 11,990 individuals. County-level areas (including counties, county-level cities, and districts) were selected as the primary sampling unit (PSU), and villages (cun) in rural areas and neighborhoods (shequ or juweihui) in urban areas were selected as the secondary sampling unit (SSU). The survey covered 476 villages within 28 out of 31 provinces in mainland China. The recruited participants were Chinese citizens aged 60 or above (no upper age limit) living at the permament address. The survey was conducted in the form of face-to-face interviews, with interviewers reading the survey’s questions and answers one by one. The interviewee selected the corresponding answer, and the interviewer recorded the answer on the questionnaire. Of the 11,990 questionnaires distributed, 11,396 were considered acceptable. Statistical analyses were conducted on a sample of 11,396 responses. The Capital University of Physical Education and Sport Ethics Committee approved the study protocol (ChiCTR-IOR-ChiCTR2200063177).

### 2.2. Measures

#### 2.2.1. PA and SB Measurements

The Chinese version of the International Physical Activity Questionnaire (IPAQ-C) incorporated in CLASS 2020 was used to collect information on PA and other factors, and it has been validated in China to measure moderate-to-vigorous PA, walking, and SB [35]. Here, physical activity included moderate-to-vigorous physical activity (MVPA) and light physical activity (LPA), which were obtained from the moderate- and vigorous-intensity and walking PA data in the IPAQ-C.

The total weekly hours (h/week) of moderate- and vigorous-intensity PA and LPA in the previous seven days were determined by asking respondents to state the hours per day (h/d) spent engaged in moderate, vigorous, and light PA. The daily hours were multiplied by 7 to calculate the h/week. The moderate and vigorous h/week were summed to identify the h/week spent engaged in moderate-to-vigorous PA. The total PA h/week was calculated by adding the weekly hours of MVPA and LPA. SB h/week was calculated by asking the respondents to estimate the h/day spent engaged in sedentary behavior (e.g., sitting, watching TV, and reading) and then multiplying this value by 7. MVPA was modified to account for rainy days, when MVPA is often not carried out. The number of rainy days was subtracted from MVPA reported for the previous seven days (MVPA in past seven days—rainy days). MVPA reported for the previous seven days was then divided by the difference (past seven days/days not raining). The average time remaining was multiplied by 7 to normalize the time spent engaged in MVPA in the previous seven days.

#### 2.2.2. Environment Measures

The environmental measures, namely, AQI, PM_2.5_, PM_10_, and nitrogen dioxide (NO_2_) (µg/m^3^), came from the Ministry of Environmental Protection of the People’s Republic of China, and daily weather data, including the daytime temperature (C), wind speed (m/s), and percentage of rainy days, came from the China Meteorological Administration (https://www.cma.gov.cn).

### 2.3. Data Analysis

Descriptive statistics, namely, means, standard deviations (±SD), and percentages, were used to summarize and compare the characteristics of the overall sample. According to the survey population’s location data, we obtained the longitude and latitude of each location using Baidu maps (https://map.baidu.com). ArcGIS was used to perform a series of visual operations on the data. The survey area did not include Hong Kong, Macao, Taiwan, Xinjiang, Tibet, or Hainan.

To facilitate the interpretation of the results, we standardized the average air pollution concentration over the previous seven days via centering (i.e., subtracting the mean from each value) and then divided it by its SD to create normalized scores (i.e., AQI z-scores). Thus, the estimated coefficient of the air pollution concentration was interpreted as the change in the outcome variable according to the changes in the air pollution concentration by one SD. Substantial variations in the air pollution concentration were present from March 2020 to November 2020. The basic z-score formula for a sample is z = (x − µ)/σ.

A data analysis was performed to evaluate the associations between AQI, MVPA, LPA, and SB. Two-sample independent t-tests were conducted on continuous variables, and chi-squared tests were conducted on dichotomous and categorical variables for the characteristics of the participants. We used multivariable linear regression adjusting for individual factors (age, education, BMI, marital status, retirement status, self-rated mental and physical health, and chronic diseases diagnosed in the previous year) and other key variables (months and regions).

The key independent variables were the AQI, PM_2.5_, PM_10_, and NO_2_ z-scores. Individual-level time-variant covariates and environmental measures, including the average daytime temperature, average wind speed, and percentage of rainy days over the previous seven days, were adjusted variables that were controlled for the aforementioned. Separate regressions were conducted for each outcome variable and based on samples stratified by sex (i.e., separate regressions were based on the entire sample with both male and female sexes).

The following individual-level time-variant covariates were variables that were controlled for in the regression analyses: a continuous variable for age in years and disease number, a continuous variable for education calculated from self-reported education received 9 years, a dichotomous variable for current retirement status (current non-retirement as the reference group), continuous variables for self-rated physical health (1–10, poor to excellent) and self-rated mental health (1–10, poor to excellent), a categorical variable for BMI (18.5 ≤ BMI < 24 kg/m², lean, normal, and overweight), and marital status (classified into married, widowed, divorced and never married). The key seasonal variables from months (survey conducted) and regions were considered in the model.

All statistical analyses were conducted using ArcGIS and Stata software, version 16 (Esri, Aylesbury, UK).

## 3. Results

### 3.1. Descriptive Statistics

Table 1 presents the characteristics of the study participants, the descriptive statistics of all the study variables for the total sample, and the descriptive statistics of all the study variables stratified by sex. Men accounted for 50.43% of the participants, while women accounted for 49.57% of the participants. The average age was 73 ± 6.61 y, and most of the participants (75.46%) had a normal weight. The average number of chronic diseases was 1.4, with women having more chronic diseases than men. Most of the participants (94%) were of Han ethnicity, and most reported fair, good, or excellent physical health (83.99%) and mental health (80.77%). The percentages of older adults who had received more than 9-year education, who were married, and who were post-retirement were 5.03%, 75.38%, and 40.71%, respectively. Among the 11,396 participants, the average times spent in MVPA, LPA, and SB were 0.8 h/week, 12.29 h/week, and 23.75 h/week, respectively. Men spent less time engaged in SB than women and more time engaged in MVPA and LPA than women.

**Figure 1:**
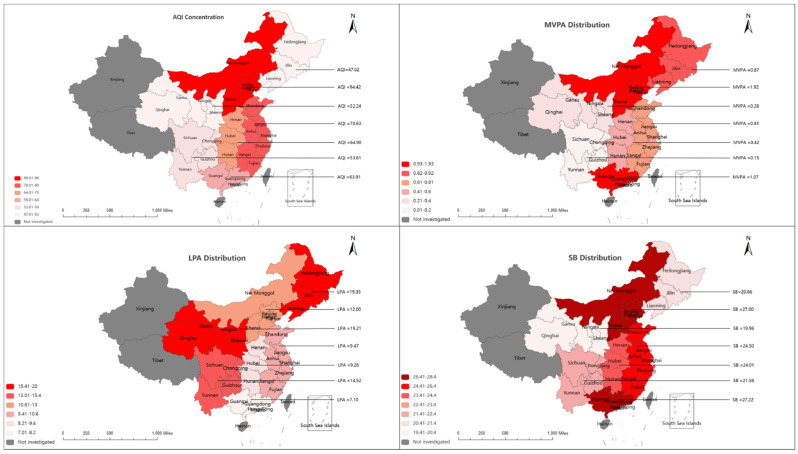
National spatial patterns of AQI, MVPA, LPA, and SB distributions in seven administrative regions in China in 2020 (the survey did not cover Xinjiang, Hainan, Tibet, Hong Kong, Macao, or Taiwan provinces/autonomous regions).

Figure 1 shows visual national spatial patterns of the average AQI concentration and MVPA, LPA, and SB distributions in the previous 7 days in seven administrative regions of China: North China (Huabei), Northeast China (Dongbei), East China (Huadong), Central China (Huazhong), South China (Huanan), Southwest China (Xinan), and Northwest China (Xibei). The AQI concentration was the highest in North China, followed by that in East China. The air pollution concentration in Northwest China was the lowest. The MVPA distribution in the older adults was the highest in North China, followed next by South China, and last by Southwest China. The average LPA distribution of the older adults was the highest in Northeast and East China. The average SB distribution was the highest in North and South China, followed next by East China.

Table 2 shows that the average AQI concentration was higher in North and East China than in the other areas, at 94.42 µg/m^3^ and 70.63 µg/m^3^, respectively. In South China and North China, the average times spent engaged in SB were 27.22 h/week and 27.00 h/week, respectively. In Northeast and Northwest China, the times spent engaged in LPA were 19.35 h/week and 19.21 h/week, respectively. The time spent engaged in MVPA was 1.92 h/week in North China, followed by 1.07 h/week in South China.

### 3.2. The Relationship between Air Pollution and MVPA, LPA, and SB

Table 3 reports the estimated effects of air pollution on individual-level MVPA outcomes by sex using multivariable linear regression. The average time spent engaged in MVPA was negatively associated with AQI, PM_2.5_, PM_10_, and NO_2_ among the 1744 participants in all the samples who reported MVPA. Increases in the SDs of the air pollution concentrations were associated with decreases in MVPA h/week as follows: AQI (−2.34, 95%CI = −3.36, −1.32), PM_2.5_ (−2.58, 95%CI = −3.61, −1.55), PM_10_ (−1.96, 95%CI = −3.05, −0.86), and NO_2_ (−1.19, 95%CI = −2.06, −0.31). Men decreased their MVPA h/week in response to AQI (−3.48), PM_2.5_ (−3.65), PM_10_ (−2.89), and NO_2_ (−1.98) concentrations. Women’s average time spent engaged in MVPA was weakly associated with air pollution.

Table 4 reports the estimated effects of air pollution on individual-level LPA outcomes by sex using multivariable linear regression. The average time spent engaged in LPA was negatively associated with AQI, PM_2.5_, PM_10_, and NO_2_ among the survey participants. Increases in the SDs of the air pollution concentrations were associated with decreases in LPA h/week as follows: AQI (−6.06, 95%CI = −7.15, −4.97), PM_2.5_ (−4.86, 95%CI = −5.88, −3.85), PM_10_ (−4.78, 95%CI = −5.89, −3.68), and NO_2_ (−4.59, 95%CI = −5.57, −3.61). Men decreased their time spent engaged in LPA in response to PM_2.5_ and PM_10_ more than women, while women decreased their time spent engaged in LPA in response to NO_2_ more than men. An analysis by sex showed that a one SD increase in air pollution was associated with a decrease in LPA h/week for both sexes for AQI (females, −5.78, 95%CI = −7.35, −4.20; males, −6.35, 95%CI = −7.87, −4.84), PM_2.5_ (females, −4.48, 95%CI = −5.94, −3.03; males, −5.21, 95%CI = −6.62, −3.81), PM_10_ (females, −4.45, 95%CI = −6.05, −2.85; males, −5.12, 95%CI = −6.65, −3.59), and NO_2_ (females, −4.79, 95%CI = −6.20, −3.38; males, −4.38, 95%CI = −5.75, −3.01).

Table 5 shows the results of the interval regression analysis between air pollution concentration and other environmental variables and SB among the participants. SB was positively associated with AQI, PM_2.5_, PM_10_, and NO_2_ among the survey participants. Increases in the SDs of the air pollution concentrations were associated with increases in SB h/week as follows: AQI (1.32 h/week, 95%CI = 0.77, 1.88), PM_2.5_ (0.62 h/week, 95%CI = 0.09, 1.14), PM_10_ (1.03 h/week, 95%CI = 0.48, 1.59), and NO_2_ (0.98 h/week, 95%CI = 0.46, 1.49). Women increased their time spent engaged in SB in response to air pollution more than men. An analysis by sex showed that a one SD increase in air pollution was associated with an increase in SB among both females and males for AQI (females, 1.54 h/week, 95%CI = 0.73, 2.34; males, (1.11 h/week, 95%CI = 0.35, 1.88); PM_2.5_ (females, 0.68 h/week, 95%CI = 0.08, 1.44; males, 0.55 h/week, 95%CI = −0.17, 1.27), PM_10_ (females, 1.27 h/week, 95%CI = 0.46, 2.09; males, 0.81 h/week, 95%CI = 0.04, 1.58), and NO_2_ (females, 0.90 h/week, 95%CI = 0.15, 1.66; males, 1.05 h/week, 95%CI = 0.34, 1.76).

## 4. Discussion

The findings from this study demonstrate the relationship of air pollution with MVPA, LPA, and SB among adults aged 60 years or older in China. Higher AQI, PM_2.5_, and PM_10_ indexes were negatively associated with the h/week spent engaged in MVPA and LPA, and they were positively associated the h/week spent engaged in SB in the total sample. Reducing ambient air pollution may improve residents’ physical behaviors by allowing them to increase their PA and decrease their SB, indirectly contributing to their health. The government should not only remind the public of air pollution’s impact on health but also take environmental measures to prevent air pollution in order to avoid changes in the older adult population’s PA and SB.

A contribution of Figure 1 is that it covers the most urban and rural cities of 30 provinces in mainland China, while its limitation is that we used the average data from the samples to represent those of all seven administrative regions. The present results given here can be used to establish a national context for studies of individual rural and urban areas. Based on their spatial distributions, the times spent engaged in LPA in Northeast China (Heilongjiang, Jilin, and Liaoning provinces) and Northwest China (Shannxi, Ningxia, and Gansu provinces) were higher than those in the other areas. One possible reason for this is that the aging population rate in most of these provinces is high (ranking at 18.7% in the country), even higher than that in other provinces. Further, adults aged 60 or older accounted for 20% of the residents in Heilongjiang, Jilin, and Liaoning provinces in 2020. The highest average time spent engaged in SB was in North China (Beijing–Tianjin–Hebei Region, Inner Mongolia, and Shanxi) and in South China (Guangdong and Guangxi), followed by other coastal areas in Eastern China. This may be due to more professional older adults being re-employed following retirement and returning to a five-day work pattern. Moreover, the time spent engaged in MVPA was high in North China and the south China coast. One possible reason for this higher time spent engaged in MVPA is the relatively developed sports industry and the higher Sports Livelihood Happiness Index in Beijing and the coastal areas [36]. These coastal areas are among the top 10 economically powerful provinces in China, and the economic development may be a vital parameter in public service for national fitness in terms of facilities and urban development. However, the inequalities in the average AQI concentration and MVPA, LPA, and SB distributions in these seven administrative regions highlight the need for future work to be carried out in order to explore the reasons.

The evidence that the impacts of climate change have dramatic consequences for human physical activity behaviors has been supported in previous studies [37]. Ambient air pollution was associated with a decline in PA in this study, which is in agreement with prior research conducted in China and in locations outside of China [23,32,38]. An et al. showed that higher ambient PM_2.5_ air pollution discourages PA among US older adults [38]. In China, Tainio et al. showed that the pooled effects of high air pollution reduced PA in children and adults [30]. The present study used data from a whole-country survey of the Chinese population aged 60 years or over. The average time spent engaged in LPA was negatively associated with air pollution in total sample. Increases in the SDs of PM_2.5_ were associated with decreases in the total hours spent engaged in LPA of 4.86 h/week. This result of the present study is consistent with that of Yu et al., whose study found that decreases in LPA of 4.7 h/week were associated with increases in PM_2.5_ concentrations among 800 to 890 university retirees in Beijing. This might be explained by previous studies that daily media alerts about air pollution may change decisions about performing outdoor physical activity among adults with asthma [39] and that these alerts are associated with shorter walking times in South Korea [40]. Moreover, home-based physical activities have been implemented [41,42], and they are popular in China [43]. Men decreased their time spent engaged in MVPA in response to AQI, PM_2.5_, and PM_10_. However, women’s average time spent in MVPA was hardly associated with AQI, PM_2.5_, or PM_10_. One possible explanation for this finding is the higher participation in MVPA among men than women and the low proportion of participation in MVPA overall. Similarly, an analysis by sex indicated a slightly weaker relationship between air pollution and SB among men and women.

Combinations of air pollution features generally explained more of the variations in physical behaviors than the single variables in terms of AQI, PM_2.5_, PM_10_, and NO_2_. An important finding was that different pollutants had slightly different associations with MVPA, LPA, and SB. Without considering rainy days, increases in the SDs of AQI, PM_2.5_, PM_10_, and NO_2_ concentrations were associated with decreases in the total hours of MVPA of 2.34, 2.58, 1.96, and 1.19 h/week, respectively. PM_2.5_ was the most salient indicator out of the air pollution concentrations, and AQI, PM_2.5_, PM_10_, and NO_2_ were hardly associated with women’s average time spent engaged in MVPA. Increases in the SDs of AQI, PM_2.5_, PM_10_, and NO_2_ concentrations were associated with decreases in the total hours of LPA of 6.06, 4.86, 4.78, and 4.59 h/week, respectively. For SB, a one SD increase in AQI, PM_2.5_, PM_10_, and NO_2_ was associated with increases in the total weekly hours of SB of 1.32, 0.62, 1.03, and 0.98 h/week, respectively. The association between PM_2.5_ and SB was weaker than that between PM_10_, NO_2_, and SB, and there is no known plausible reason for this finding. However, it provides direction for future climate-related physical behavior studies to take particles other than PM_2.5_ as variables.

When the novel coronavirus disease (COVID-19) outbroke, China launched unprecedented control measures. Under joint prevention and control policies, on 7 March 2020, 44 new cases of confirmed infections were reported, with very few local community-based cases in China outside of Hubei province [44]. Apart from those who were home-isolating, all individuals were asked to be hospitalized in square cabin hospitals, and all close contacts and suspected cases were required to complete mandatory isolation in special facilities. People, including older adults wearing masks, could participate in outdoor PA and walk outside in communities and parks after scanning their health code and registering. This study has notable limitations associated with the selection of the IPAQ-C for assessing PA participation. The instrument could be more precise in measuring the duration and intensity of PA compared with the information frequency of PA. However, this limitation was balanced with a need to select a robust and easily administrated instrument that has been widely used in related epidemiology literature and that has been validated in the Chinese population. More objective methods, such as accelerometers, could be applied to assess PA and SB; however, they are not practical in large population studies. Moreover, this study did not include Xinjiang, Tibet, Hainan Island, or Taiwan populations. Finally, the findings regarding sex differences in relation to air pollution and PA are somewhat preliminary and warrant replication in future research. Limitations aside, our study nonetheless provides a deep insight into the relationships between AQI, PM_2.5_, PM_10_, and NO_2_ concentrations and PA and SB among large samples of Chinese older adults selected from major provinces across China.

## 5. Conclusions

The spatial distributions of the average AQI concentration, MVPA, LPA, and SB are useful and allow environmental and health policymakers to identify the areas with the highest priority air pollution environmental equality concerns. AQI was positively associated with MVPA and LPA, and it was negatively associated with SB among older adults. AQI, PM_2.5_, PM_10_, and NO_2_ were hardly associated with women’s average time spent engaged in MVPA. Region-specific and multi-level health policy options are needed to reduce ambient air pollution by taking different types of pollutants into account in order to avoid changes in PA and SB in this population, especially in locations with high air pollution concentrations.

## Figures and Tables

**Table 1:** Characteristics of survey participants (n = 11,396).

Variable			
	Men	Women	Total
Sex, n (%)	5747 (50.43)	5649 (49.57)	11,396 (100)
Age (y), mean (SD)	73.50 (6.38)	73.67(6.82)	73.59 (6.61)
Nationality, n (%)			
Han	5389 (47.29)	5325 (46.73)	10,714 (94.02)
Minority groups	358 (3.14)	324 (2.84)	682 (5.98)
BMI, n (%) ^a^			
Lean	302(2.65)	294(2.58)	596 (5.23)
Normal	4471 (39.23)	4128 (36.22)	8599 (75.46)
Overweight	974 (8.55)	1227(10.77)	2201(19.31)
Education ≤ 9 years, n (%) ^a^	573 (5.03)	390 (3.42)	963 (8.45)
Marital status, n (%)			
Married	4759 (41.76)	3831 (33.62)	8590 (75.38)
Widowed	870 (7.63)	1747 (15.33)	2617 (22.96)
Divorced/separated	56 (0.49)	53(0.47)	109(0.96)
Never married	62 (0.54)	18 (0.16)	80 (0.7)
Retirement status n (%)			
Yes	2359 (20.70)	2280 (20.01)	4639 (40.71)
No	3388 (29.73)	3369 (29.56)	6757(59.29)
Smoking, n (%)	2784 (24.43)	390 (3.42)	3174 (27.85)
Physical health, n (%)	5747 (50.43)	5649 (49.57)	11,396 (100.0)
Fair, good, or excellent health	4943 (43.37)	4629 (40.62)	9572 (83.99)
Poor or very poor health	794 (6.97)	1013 (8.89)	1807 (15.86)
No answer	10 (0.09)	7 (0.06)	17 (0.15)
Mental health, n (%)	5747 (50.43)	5649 (49.57)	11,396 (100.0)
Good or excellent	4645 (40.76)	4559 (40.01)	9204 (80.77)
Poor	883 (7.75)	870 (7.63)	1753 (15.38)
No answer	219 (1.92)	220 (1.93)	439 (3.85)
Disease number, mean (SD)	1.31 (1.36)	1.50 (1.48)	1.40 (1.42)
PA and SB			
MVPA (h/week), mean (SD) ^a^	0.87 (2.91)	0.73 (2.43)	0.80 (2.68)
LPA (h/week), Mean (SD) ^a^	12.48 (10.83)	12.09 (10.62)	12.29 (10.73)
SB (h/week), mean (SD) ^a^	23.37 (12.30)	24.13 (12.95)	23.75 (12.63)

^a^ BMI = body mass index (18.5 ≤ BMI < 24 kg/m²), MVPA = moderate-to-vigorous physical activity, LPA = light physical activity, SB = sedentary behavior, education = 9 years of high school until graduation.

**Table 2:** Average time spent engaged in MVPA, LPA, and SB and air pollution concentrations in the previous seven days before the survey (n = 11,396) in seven administrative regions of China.

Zones ^a^	Huabei	Dongbei	Huadong	Huazhong	Huanan	Xinan	Xibei
Dependent variables	n = 1558	n = 1531	n = 3359	n = 1721	n = 876	n = 1585	n = 766
MVPA (h/week), mean (SD)	1.92 (3.55)	0.87 (2.90)	0.81 (2.85)	0.42 (2.74)	1.07 (2.09)	0.15 (0.95)	0.28 (1.16)
LPA (h/week), mean (SD)	12.00 (9.16)	19.35 (8.54)	9.47 (10.08)	9.26 (10.77)	7.10 (9.28)	14.52 (11.26)	19.21 (9.16)
SB (h/week), mean (SD)	27.00 (15.68)	20.66 (7.80)	24.50 (15.94)	24.01 (10.39)	27.22 (10.06)	21.58 (8.58)	19.98 (6.87)
Air pollution							
AQI (µg/m^3^), mean (SD)	94.42 (48.69)	47.02 (14.38)	70.63 (26.80)	64.90 (23.35)	63.91 (14.14)	53.61 (18.42)	52.24 (11.16)
PM_2.5_ (µg/m^3^), mean (SD)	46.98 (23.59)	23.71 (14.49)	29.61 (13.23)	34.56 (19.46)	29.20 (11.64)	27.60 (15.31)	21.54 (5.97)
PM_10_ (µg/m^3^), mean (SD)	110.94 (74.20)	46.29 (20.13)	66.67 (47.41)	57.59 (27.02)	57.32 (24.14)	44.67 (24.04)	47.11 (16.25)
NO_2_ (µg/m^3^), mean (SD)	34.77 (11.25)	25.92 (8.77)	26.80 (10.71)	21.94 (8.88)	25.78 (9.17)	21.63 (7.64)	28.35 (14.61)
Environmental covariates							
Temperature (°C), mean (SD)	16.60 (47.07)	9.03 (6.44)	20.27 (5.12)	20.30 (4.05)	23.75 (2.36)	18.28 (4.87)	11.86 (6.85)
Sun (day/week), mean (SD)	2.31 (1.35)	1.97 (1.81)	1.04 (1.22)	0.96 (1.33)	0.92 (1.68)	1.52 (1.86)	1.27 (1.69)
Rain (day/week), mean (SD)	1.44 (1.58)	1.95 (1.56)	2.89 (2.07)	3.18 (1.88)	1.98 (1.50)	3.02 (2.02)	3.04 (1.90)

^a^ Zones (Seven administrative regions of China) refer to Huabei (Hebei, Beijing, Tianjin, Shanxi, Inner Mongolia autonomous zone), Dongbei (Heilongjiang, Jilin, Liaoning provinces), Huadong (Shandong, Jiangsu, Shanghai, Zhejiang, Anhui, Jiangxi, Taiwan provinces), Huazhong (Henan, Hubei, Hunan provinces), Huanan (Guangdong, Guangxi autonomous zone, Hainan, Hong Kong, and Macao), Xinan (Sichuan, Yunnan, Guizhou, Chongqing, Tibet autonomous zone), and Xibei (Xinjiang autonomous zone, Ningxia autonomous zone, Qinghai, Shanxi, Gansu provinces).

**Table 3:** Association of air pollution with individual-level MVPA outcomes by sex (n = 1744).

Dependent Variable	Men		Women		Total	
	Coefficient (95%CI)	n	Coefficient (95%CI)	n	Coefficient (95%CI)	n
AQI Moderate and Vigorous PA (h/week)	−3.48 *** (−5.19, −1.76)	912	−1.11 * (−2.11, −0.12)	832	−2.34 *** (−3.36, −1.32)	1744
PM_2.5_ Moderate and Vigorous PA (h/week)	−3.65 *** (−5.37, −1.93)	912	−1.44 ** (−2.47, −0.42)	832	−2.58 *** (−3.61, −1.55)	1744
PM_10_ Moderate and Vigorous PA (h/week)	−2.89 *** (−4.73, −1.05)	912	−1.03 (−2.10, −0.04)	832	−1.96 *** (−3.05, −0.86)	1744
NO_2_ Moderate and Vigorous PA (h/week)	−1.98 ** (−3.48, −0.49)	912	−0.29 (−1.14, 0.55)	832	−1.19 ** (−2.06, −0.31)	1744

* *p* < 0.05; ** *p* < 0.01; *** *p* < 0.001.

**Table 4:** Association of air pollution with individual-level LPA outcomes by sex (n = 8604).

Dependent Variable	Men		Women		Total	
	Coefficient (95%CI)	n	Coefficient (95%CI)	n	Coefficient (95%CI)	n
AQI LPA(h/week)	−6.35 *** (−7.87, −4.84)	4342	−5.78 *** (−7.35, −4.20)	4262	−6.06 *** (−7.15, −4.97)	8604
PM_2.5_ LPA(h/week)	−5.21 *** (−6.62, −3.81)	4342	−4.48 *** (−5.94, −3.03)	4262	−4.86 *** (−5.88, −3.85)	8604
PM_10_ LPA(h/week)	−5.12 *** (−6.65, −3.59)	4342	−4.45 *** (−6.05, −2.85)	4262	−4.78 *** (−5.89, −3.68)	8604
NO_2_ LPA(h/week)	−4.38 *** (−5.75, −3.01)	4342	−4.79 *** (−6.20, −3.38)	4262	−4.59 *** (−5.57, −3.61)	8604

*** *p* < 0.001.

**Table 5:** Associatioin of air pollution with individual-level sedentary behavior outcomes by sex (n = 11,396).

Dependent Variable	Men		Women		Total	
	Coefficient (95%CI)	n	Coefficient (95%CI)	n	Coefficient (95%CI)	n
AQI Sedentary Behavior (h/week)	1.11 ** (0.35, 1.88)	5747	1.54 *** (0.73, 2.34)	5649	1.32 *** (0.77, 1.88)	11,396
PM_2.5_ Sedentary Behavior (h/week)	0.55 (−0.17, 1.27)	5747	0.68 * (0.08, 1.44)	5649	0.62 * (0.09, 1.14)	11,396
PM_10_ Sedentary Behavior (h/week)	0.81 * (0.04, 1.58)	5747	1.27 *** (0.46, 2.09)	5649	1.03 *** (0.48, 1.59)	11,396
NO_2_ Sedentary Behavior (h/week)	1.05 ** (0.34, 1.76)	5747	0.90 * (0.15, 1.66)	5649	0.98 *** (0.46, 1.49)	11,396

* *p* < 0.05; ** *p* < 0.01; *** *p* < 0.001.

## Data Availability

The datasets from the current study are available from the corresponding author upon reasonable request.
